# Silicone ring tourniquet versus pneumatic cuff tourniquet in carpal tunnel release: a randomized comparative study

**DOI:** 10.1007/s10195-012-0223-x

**Published:** 2013-01-30

**Authors:** G. I. Drosos, A. Ververidis, N. I. Stavropoulos, R. Mavropoulos, G. Tripsianis, K. Kazakos

**Affiliations:** 1 Department of Orthopaedic Surgery, Medical School, University General Hospital of Alexandroupolis, Democritus University of Thrace, 68100 Alexandroupolis, Greece; 2 General Hospital of Kalamata, Kalamata, Greece; 3 Department of Medical Statistics, Medical School, Democritus University of Thrace, 68100 Alexandroupolis, Greece

**Keywords:** Tourniquet, Pneumatic tourniquet, Silicon ring tourniquet, Tourniquet pain

## Abstract

**Background:**

The aim of the present study was to compare the pain levels resulting from the use of a silicone ring tourniquet (SRT) to those resulting from the use of a classic pneumatic cuff tourniquet (PT) in patients undergoing carpal tunnel release under local anesthesia.

**Materials and methods:**

Fifty patients that underwent carpal tunnel release under local anesthesia were randomized using the technique of stratified randomization by minimization. A forearm tourniquet was applied: a standard PT was used in 25 patients, and an SRT was used in the other 25 patients (the model of SRT used was selected according to the standard systolic blood pressure). Patient demographics and complications were recorded. Pain levels were assessed with the visual analogue scale and were recorded (a) just after tourniquet application, (b) 5 min after tourniquet application, and (c) just before tourniquet removal.

**Results:**

There was no statistical significant difference in patient demographics between the two groups. The mean tourniquet time was similar for both groups (*p* = 1.000). The difference between the mean final pain level and the mean initial pain level was statistically significant for the SRT group (*p* = 0.010) and highly statistically significant for the PT group (*p* < 0.001). The mean final pain level for the PT group was higher than that for the SRT group (*p* = 0.043).

**Conclusions:**

According to the findings of this study, in patients who underwent carpal tunnel release under local anesthesia, the pain levels at the end of the operation and those just before the removal of the tourniquet were higher in the PT group than in the SRT group of patients.

## Introduction

Tourniquet devices are commonly used in orthopedic procedures in order to provide a bloodless operating field during surgical procedures involving the extremities. Pneumatic tourniquets (PTs) are preferred by most surgeons, and modern pneumatic tourniquets are designed to minimize the incidence of complications [1]. Nevertheless, complications do still occur, and a recent study showed that the incidence of tourniquet complications is still at least as high as that estimated in the 1970s [2].

However, a new device known as a silicon ring tourniquet (SRT) was introduced into clinical practice relatively recently [3–6]. This a novel device (marketed as the S-MART or HemaClear, OHK Medical Devices, Haifa, Israel) consists of a silicone ring wrapped within an elastic sleeve (stockinet) and two straps attached to pull handles, and is designed for exsanguination and occlusion of the blood flow to the limb.

The entire device is sterile and comes in different sizes: (a) a small size for pediatric use; (b) a medium size for an upper limb (circumference of the limb at the occlusion site 24–40 cm); (c) a large size for the leg (circumference of the limb at the occlusion site 30–55 cm); and (d) an extra-large size for the leg (circumference of the limb at the occlusion site 50–90 cm and systolic blood pressure ≤160 mmHg). There are three tension models (systolic blood pressure ≤130 mmHg, <160 mmHg, <190 mmHg) for the medium and large sizes, and the appropriate model is selected for each patient according to the systolic blood pressure measured in the operating room before the placement of the device.

This device has been compared to the pneumatic tourniquet in healthy volunteers [7, 8], and in a clinical study of patients that underwent upper extremity operations [4].

The aim of this randomized prospective study was to compare the pain levels resulting from the use of the classic pneumatic tourniquet to the pain levels resulting from the use of this new device in patients undergoing carpal tunnel release.

## Materials and methods

### Study design

Patients who were scheduled for carpal tunnel release under local anesthesia and had no previous fracture or operation in the affected limb as well as no history of anemia, malignancy, or neurological disorder were included in this study. All of the patients gave their informed consent prior to being included into the study. The study was authorized by the local ethical committee (hospital ethics committee approval: UGHA 157/2-7-2010), and was performed in accordance with the ethical standards of the 1964 Declaration of Helsinki, as revised in 2000.

Fifty patients were randomized using the technique of stratified randomization by minimization [9, 10]. The patients were assigned to a treatment group (SRT or PT) according to a stratified and blocked randomization method. The randomization was based on four parameters: age (30–39, 40–49, 50–59 years), gender (male, female), body mass index (BMI) (less than 25, 25–29.9, more than 30), and whether the patient was a smoker (yes or no). Each patient’s age, gender, body mass index (BMI), smoking status, occupation, other illnesses, medications, and dominant hand were recorded. Patients were followed up for any complications related to tourniquet use or to the operation up to 30 days postoperatively.

### Tourniquet types

A standard pneumatic tourniquet (PT) with an 8 cm wide cuff and the appropriate SRT model (selected according to the standard systolic pressure) were used.

### Procedure

The procedure was explained to all participants. They were also instructed how to use the visual analogue scale for discomfort/pain (0 = no discomfort/pain; 10 = the worst pain) [11]. The patients were placed in a comfortable, supine position out of sight of clocks or monitoring equipment. The systolic and diastolic blood pressures and the pulse rate were monitored using a noninvasive monitor, and the cuff/cables were applied to the unaffected upper limb. After a 10 min period to allow the values of the recorded variables to stabilize, the systolic pressure was measured and used as the standard.

A forearm tourniquet was used in all patients. A PT with an 8 cm wide cuff was applied over two layers of smoothly applied cast padding. The limb was elevated for 3 min before tourniquet inflation. The PT inflation pressure was 100 mm Hg above the standard systolic blood pressure. The appropriate SRT model was selected according to the standard systolic blood pressure and applied as recommended by the manufacturer.

The VAS levels for pain were recorded (a) just after tourniquet application (initial pain, *T*_0_), (b) 5 min after the tourniquet application (*T*_5_), and (c) just before the tourniquet removal (final pain, *T*_final_).

The surgical technique employed was the same in all patients. Open carpal tunnel release was performed under local anesthesia with ropivacaine. A curved skin incision was made ulnar to and parallel to the thenar crease, followed by an inline incision of the subcutaneous tissue and the palmar aponeurosis. The distal end of the transverse carpal ligament was identified and the ligament was incised. The flexor tenosynovium was inspected before the skin closure with 3-0 nylon suture. A compressive dressing was applied and the hand was kept elevated for two days, during which time the patients were instructed to perform active finger movements. Subsequently, a smaller dressing was applied and the patient was encouraged to gradually resume normal use of the hand. The sutures were removed after 12–14 days.

### Statistical analysis

Statistical analysis of the data was performed using the Statistical Package for the Social Sciences (SPSS), version 19.0 (SPSS, Inc., Chicago, IL, USA). All quantitative variables were expressed as the mean ± SD, while qualitative variables were expressed as frequencies (and percentages). The normality of the quantitative variables was tested with the Kolmogorov–Smirnov test. The *χ*^2^ test and Student’s *t* test were used to assess differences in demographic characteristics between the two groups of patients. Between-group differences in VAS score were assessed by Student’s *t* test, while within-group differences were examined by one-way repeated-measures ANOVA (rmANOVA); post hoc analysis was performed using Bonferroni’s correction. The interaction between the type of the tourniquet and the change in VAS score over time was established by performing two-way analysis of variance. All tests were two-tailed and statistical significance was considered for *p* values of less than 0.05.

## Results

### Patients

There were no complications related to the tourniquet in either group, or wound infections. There were no statistically significant differences in gender (*p* = 0.713), age (*p* = 0.658), BMI (*p* = 0.712), smoking status (*p* = 1.000), occupation (*p* = 0.758), other illness (*p* = 0.569), medication (*p* = 0.569), or the dominant hand (*p* = 0.208) between the two groups of patients (Table 1).

**Table 1:** Patient demographics and pain scores (VAS)

	SRT	PT	*p* value
*N*	25	25	
Gender male [no (%)]	5 (20.0)	4 (16.0)	0.713
Age (years; mean ± SD)	54.28 ± 11.17	55.56 ± 9.06	0.658
BMI (mean ± SD)	29.08 ± 5.35	29.57 ± 3.89	0.712
Smoking status [no (%)]	7 (28.0)	7 (28.0)	1.000
Manual work (no %)	18 (72.0)	17 (68.0)	0.758
Other illness [no (%)]	13 (52.0)	15 (60.0)	0.569
Medication [no (%)]	13 (52.0)	15 (60.0)	0.569
Dominant hand right [no (%)]	20 (80.0)	16 (64.0)	0.208
Tourniquet time (min; mean ± SD)	10.20 ± 2.78	10.20 ± 3.58	1.000
VAS score (mean ± SE)			
*T*_0_	3.92 ± 2.12	3.12 ± 2.05	0.181
*T*_5_	4.44 ± 1.80	3.88 ± 1.92	0.294
*T*_final_	4.96 ± 1.65	5.88 ± 1.48	0.043
Change in VAS score			
From *T*_0_ to *T*_5_	0.52 ± 1.56 [13.3 %]	0.76 ± 1.96 [24.4 %]	0.634
From *T*_5_ to *T*_final_	0.52 ± 1.33 [11.7 %]	2.00 ± 1.83 [51.5 %]	0.002
From *T*_0_ to *T*_final_	1.04 ± 1.59 [26.5 %]	2.76 ± 2.05 [88.5 %]	0.002

*SRT* silicone ring tourniquet, *PT* pneumatic tourniquet, *BMI* bone mass index, *VAS* visual analogue scale

*T*_0_ pain level after tourniquet application, *T*_5_ pain level 5 min after tourniquet application, *T*_final_ pain level just before tourniquet removal

### Tourniquet time

The mean tourniquet time was identical in the two groups (*p* = 1.000): 10.20 ± 2.78 min (median time, 10 min) for SRT and 10.20 ± 3.58 min (median time, 10 min) for PT.

### Pain levels

**Fig. 1:**
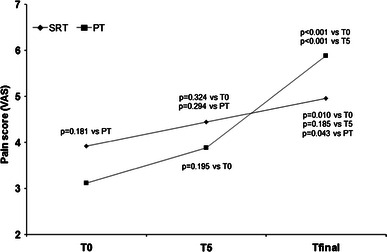
Mean pain scores over time. *SRT* silicone ring tourniquet, *PT* pneumatic tourniquet

The VAS score for pain at the site of tourniquet application is shown for each device in Table 1 and Fig. 1. Since the Kolmogorov–Smirnov test did not show any significant deviation from the normal distribution, the VAS score was expressed as the mean ± SD.

One-way repeated-measures ANOVA showed statistically significant changes in the VAS score over time (SRT: *F*_2,48_ = 6.030, *p* = 0.005; PT: *F*_2,48_ = 26.791, *p* < 0.001).

Post hoc analysis, using Bonferroni’s adjustment for the number of comparisons, was then performed: SRT application produced a gradual elevation of the VAS score from one measurement to the next by 13.3 and 11.7 %, respectively, but none of these changes reached statistical significance (*p* = 0.324 and *p* = 0.185, respectively). Overall, SRT application resulted in a statistically significant elevation of the pain score by 26.5 % (*p* = 0.010) compared to the initial pain score (from *T*_0_ to *T*_final_). In the PT group, an initial nonsignificant (*p* = 0.195) elevation of the VAS score by 24.4 % (from *T*_0_ to *T*_5_) was followed by a statistically significant elevation of the VAS score by 51.5 % (*p* < 0.001) (from *T*_5_ to *T*_final_). Overall, PT application produced a highly significant elevation of the pain score by 88.5 % (*p* < 0.001) compared to the initial pain score (from *T*_0_ to *T*_final_).

The time courses for the pain experienced by the two groups were compared via two-way mixed ANOVA. This analysis revealed a statistically significant interaction between the type of the tourniquet and the change in VAS score over time (*F*_2,48_ = 7.189, *p* = 0.001). In this regard, although the initial elevation of the VAS score was similar for the two groups (13.3 % in SRT vs. 24.4 % in PT, *p* = 0.634), the pain increased more dramatically in the PT than in the SRT group from the second measurement until tourniquet removal (11.7 % in SRT vs. 51.5 % in PT, *p* = 0.002). In addition, the increase in the pain score from tourniquet application until removal was greater in the PT than in the SRT group (26.5 % in SRT vs. 88.5 % in PT, *p* = 0.002).

A comparison of the post-application VAS scores of the two groups of patients at each measurement showed that there was: (1) no statistically significant difference between the PT and SRT groups just after tourniquet application (*p* = 0.181) and 5 min after this application (*p* = 0.294); (2) a significantly higher VAS score for the PT than for the SRT group just before tourniquet removal (*p* = 0.043).

## Discussion

According to the findings of this study, patients that underwent carpal tunnel release under local anesthesia and with the use of a forearm pneumatic tourniquet experienced more pain at the end of the procedure compared to patients for whom the silicone ring tourniquet was used. The pain levels just after the application of the tourniquet were higher in the SRT group and remained higher 5 min later when compared to the PT group. After that, the pain levels in the SRT group continued to gradually increase in the same manner as they did during the first 5 min. However, for the PT group, a more rapid increase in pain levels was observed, and the mean pain levels in the PT group had become higher than those in the SRT group by about 6–7 min after the application of the tourniquet. These fluctuations in pain levels are similar to those seen in a recently reported study of healthy volunteers [7].

A forearm tourniquet was used in our study because, according to previous studies, a forearm tourniquet is tolerated for longer than an upper arm tourniquet [8, 12].

According to previously published studies [3–6], the SRT has several advantages: it is easily applied (thus decreasing the time and effort required for tourniquet application); it is sterile and can be applied intraoperatively (saving tourniquet time); no additional step for limb exsanguination is required; and it covers a narrow area of the limb. On the other hand, the pressure applied by the SRT is fixed (cannot be adjusted), and the SRT cannot entirely replace the PT since it cannot be used in very obese patients due to limitations on the limb circumference. Furthermore, the SRT is disposable, so there is a direct cost. On the other hand, there is an indirect cost for PTs. The device requires regular maintenance, repairs, and replacements, as well as routine checking, daily calibration checks of all valves and gauges, intraoperative monitoring of tourniquet function at frequent intervals, and rigorous monthly performance-assurance tests [1].

Two recently reported studies of healthy volunteers showed that the SRT performs similarly to the classic PT in terms of tolerance time [7], and may be more comfortable than the PT when used on the upper arm [8].

The etiology and the neural pathways of tourniquet pain seem to be multifactorial [13]. The pressure applied by the tourniquet is certainly one of the responsible factors. Narrow cuffs require a higher arterial flow occlusion pressure [14, 15], and this theoretically increases the chance of pressure-related complications. Furthermore, using a non-pneumatic tourniquet for extended periods may increase the incidence of tourniquet-related adverse events. Nevertheless, such complications have not been reported in published clinical series, where the SRT was used for up to 1.5–2 h [3–6].

On the other hand, a study of human volunteers showed that narrow cuffs resulted in less pain and were tolerated for a longer time than wider cuffs [16] and, more recently, nerve conduction studies showed that wider cuffs result in more severe nerve changes than narrow cuffs inflated to the same pressure and used for the same period of time [17]. These findings may explain the results of our study.

In conclusion, according to the findings of this study, in patients who underwent carpal tunnel release under local anesthesia, the pain levels at the end of the operation and those just before the removal of the tourniquet were higher in the PT group than in the SRT group of patients. Therefore, it seems that SRT may be advantageous compared to the classic pneumatic tourniquet from a tourniquet pain perspective in hand operations performed under local anesthesia, such as carpal tunnel release.
